# Supplementary material for: Investigating the relationship between breast cancer risk factors and an AI-generated mammographic texture feature in the Nurses’ Health Study II
Source: medRxiv. 2025 Feb 20:2025.02.18.25322419. Preprint. [Version 1] doi: 10.1101/2025.02.18.25322419 (PMC11875271; doi:10.1101/2025.02.18.25322419)
Supplement: Supplement 1 [file media-1.pdf]

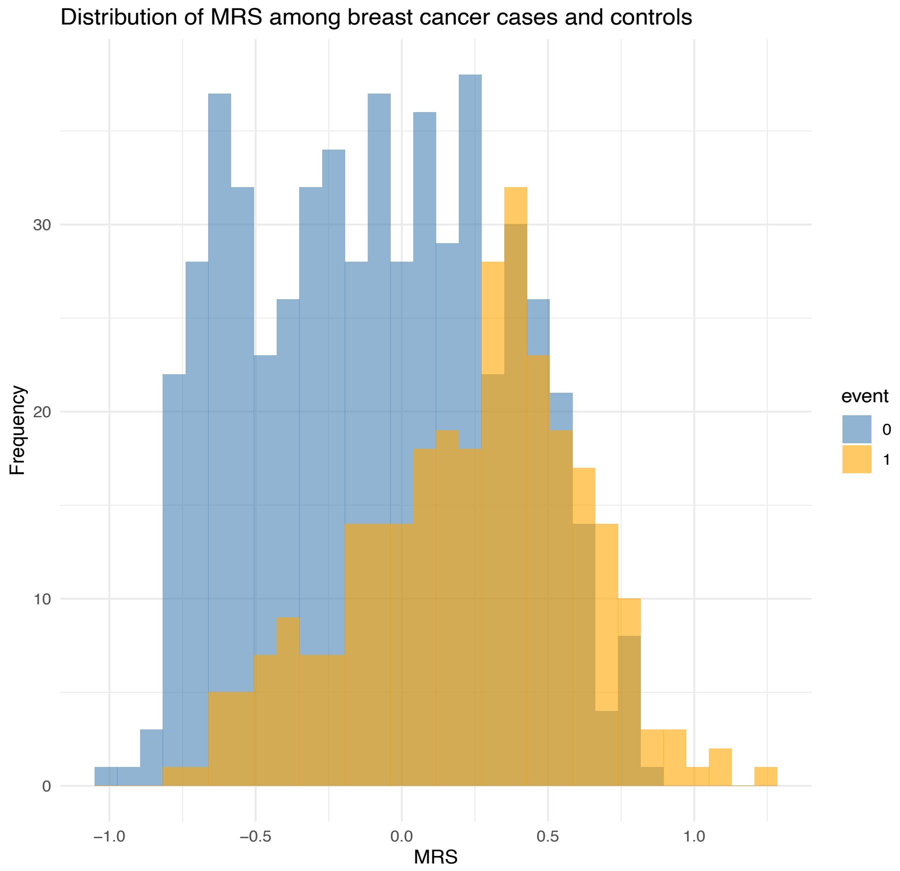

**Figure S1.** Distribution of mammogram risk score (MRS) among breast cancer cases and controls in NHS II.

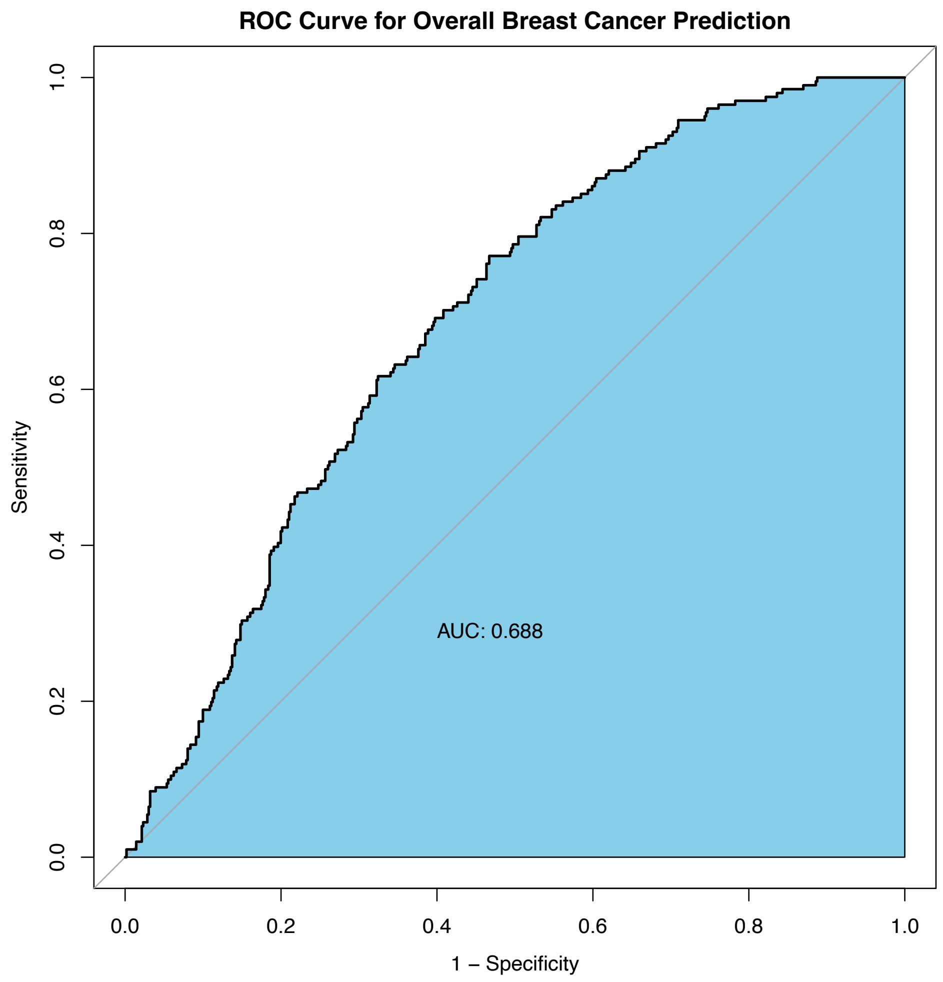


**Figure S2.** Evaluating the performance of mammogram risk score in breast cancer risk prediction in NHS II.

ROC, receiver operating characteristic. AUC, area under the ROC curve.

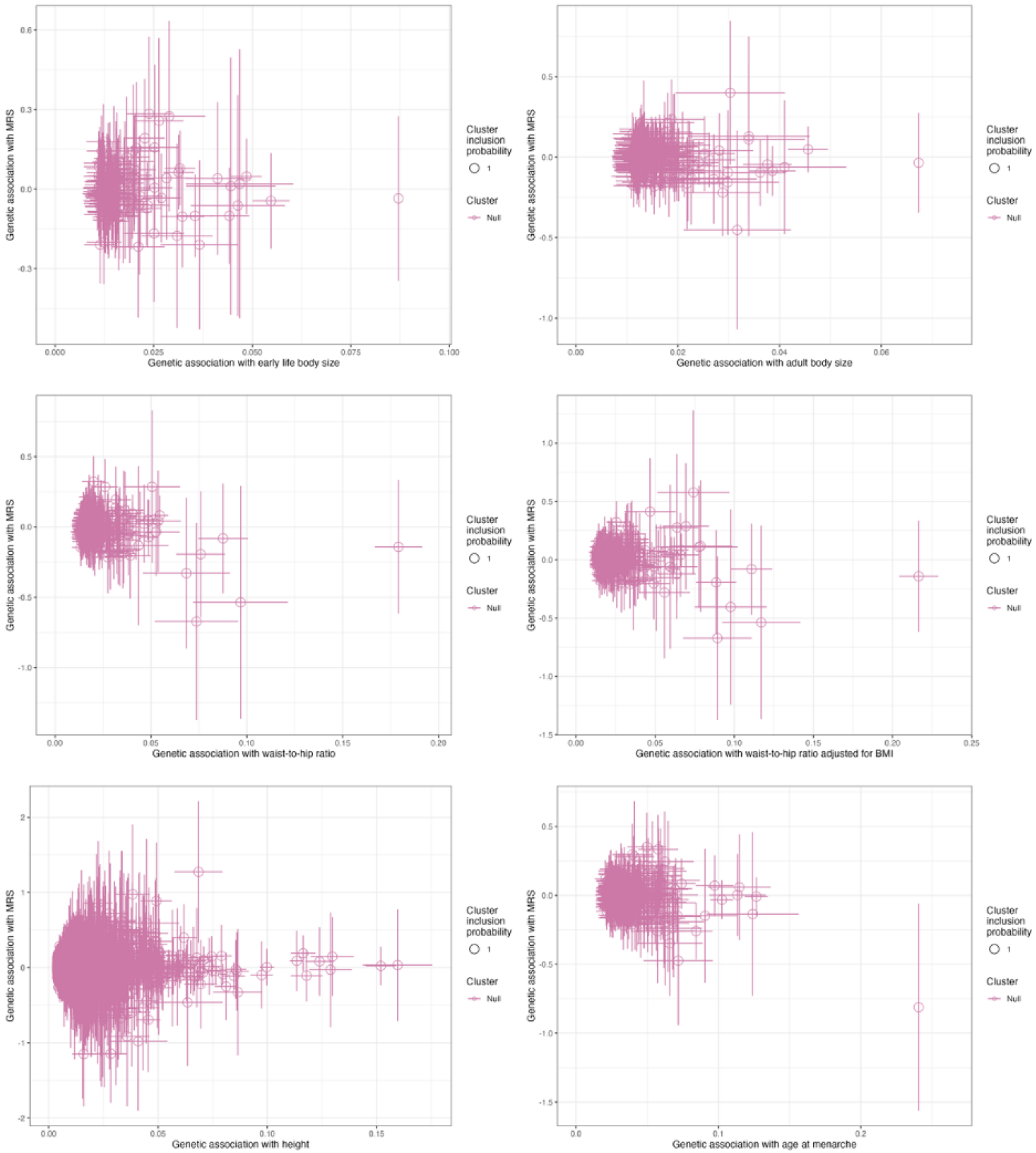


**Figure S3.** Genetic associations between each risk factor and mammogram risk score (MRS). Scatter plots show results from MR-Clust analysis, with genetic association with MRS (y-axis) versus genetic association with each risk factor (x-axis). Each point represents a genetic variant, with lines indicating 95% confidence intervals.

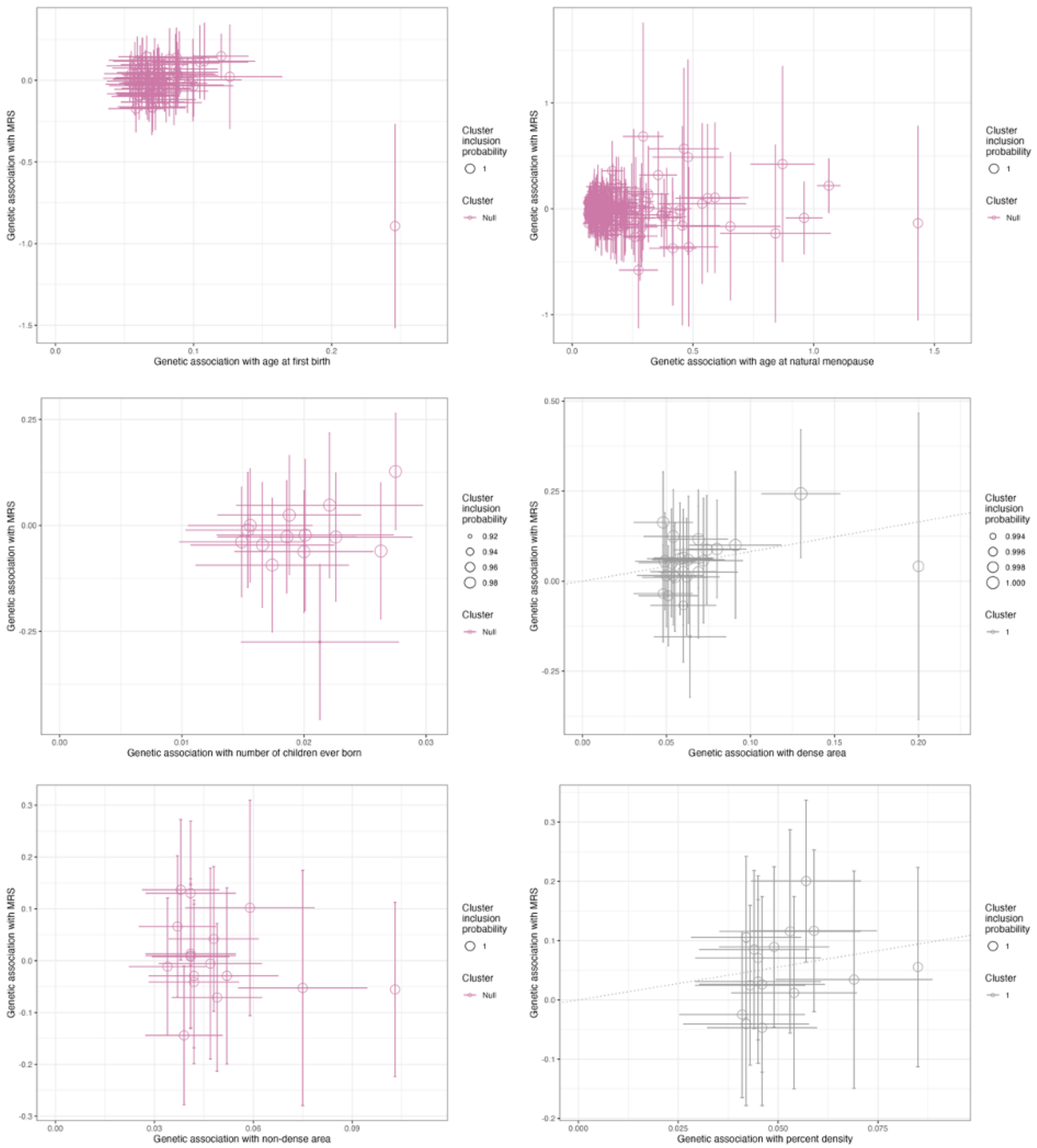


**Figure S3 (continued).** Genetic associations between each risk factor and mammogram risk score (MRS). Scatter plots show results from MR-Clust analysis, with genetic association with MRS (y-axis) versus genetic association with each risk factor (x-axis). Each point represents a genetic variant, with lines indicating 95% confidence intervals.

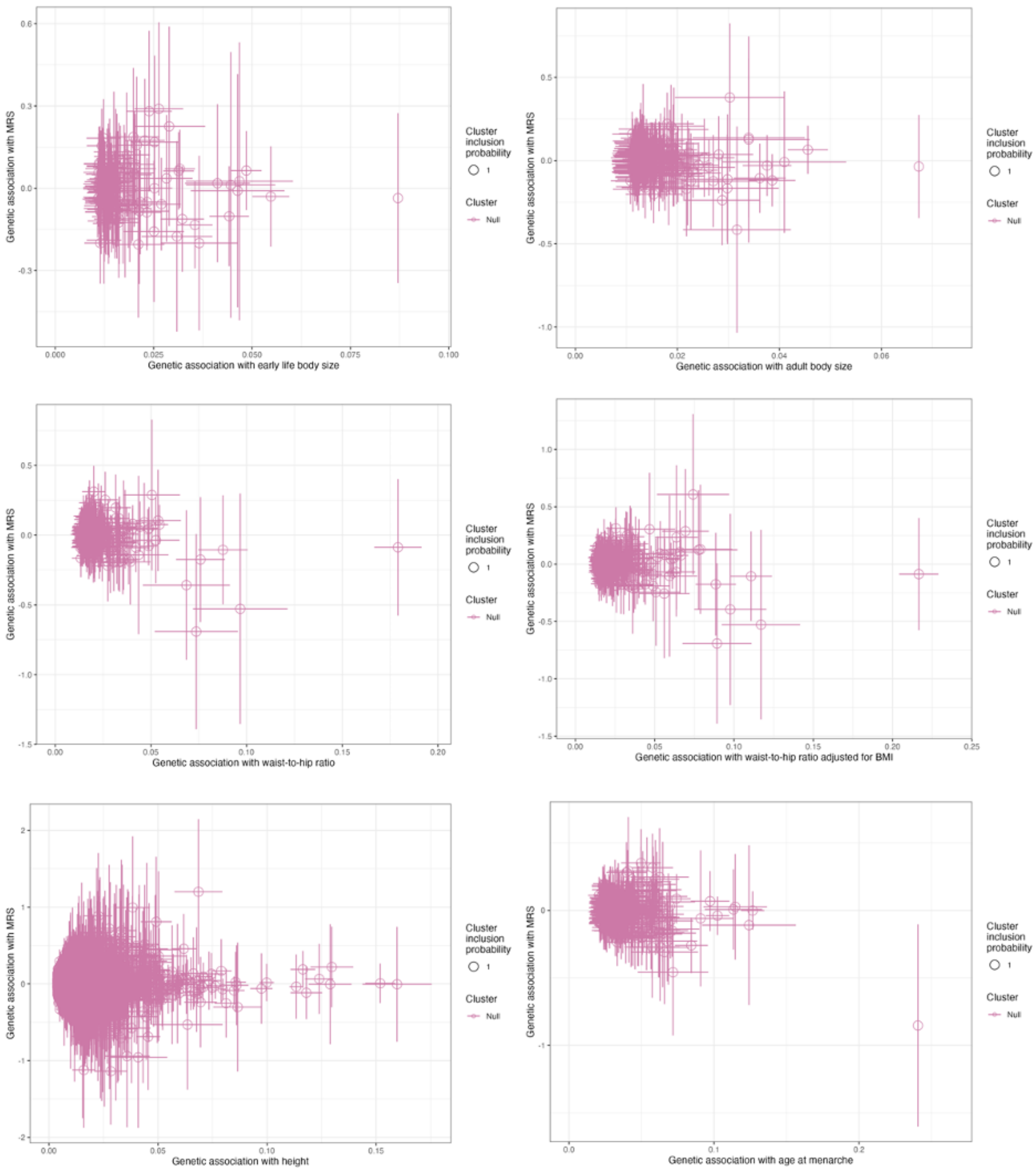


**Figure S4.** Genetic associations between each risk factor and mammogram risk score (MRS) using instrumental variable-MRS associations adjusted for menopausal status. Scatter plots show results from MR-Clust analysis, with genetic association with MRS (y-axis) versus genetic association with each risk factor (x-axis). Each point represents a genetic variant, with lines indicating 95% confidence intervals.

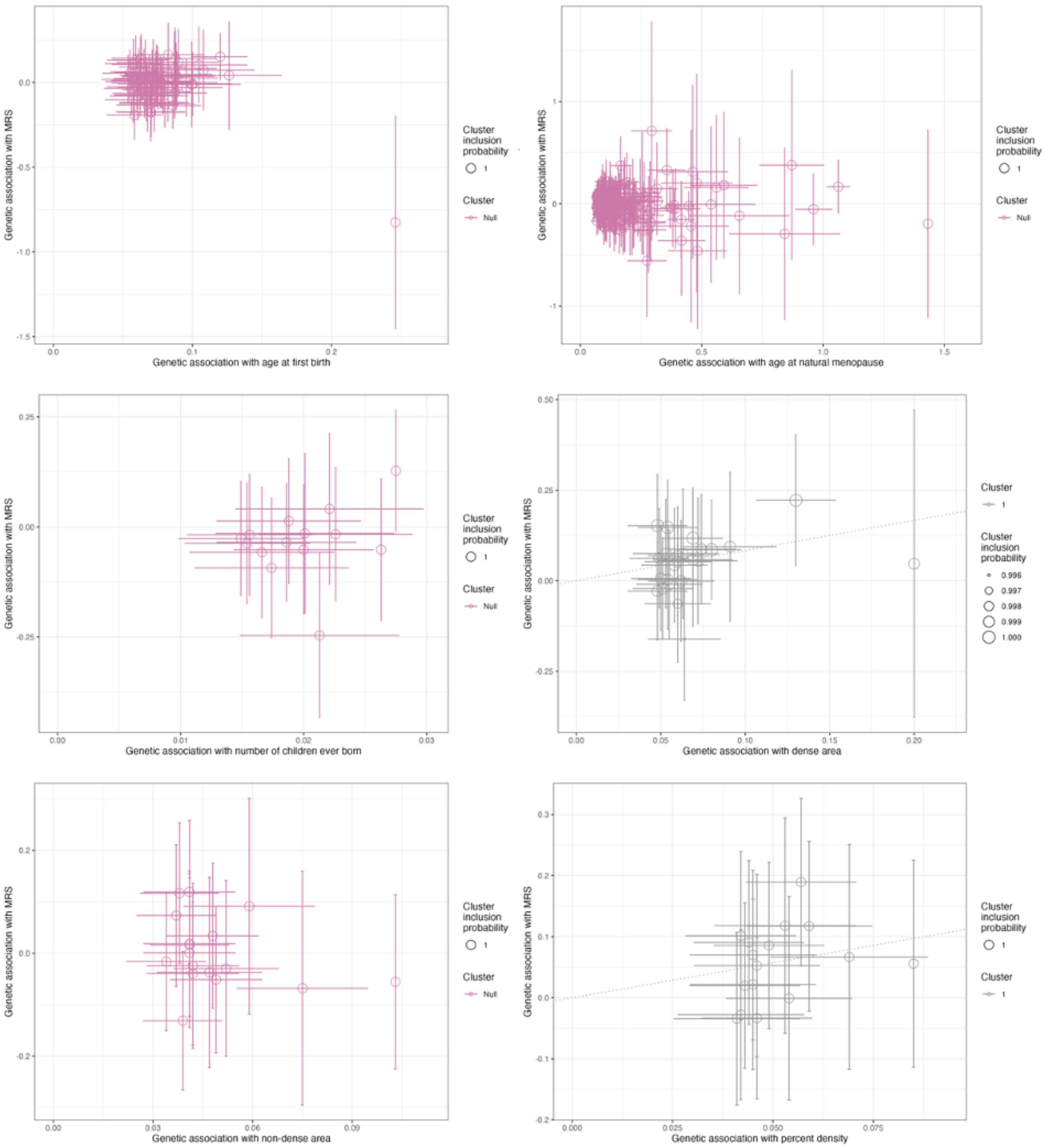


**Figure S4 (continued).** Genetic associations between each risk factor and mammogram risk score (MRS) using instrumental variable-MRS associations adjusted for menopausal status. Scatter plots show results from MR-Clust analysis, with genetic association with MRS (y-axis) versus genetic association with each risk factor (x-axis). Each point represents a genetic variant, with lines indicating 95% confidence intervals.

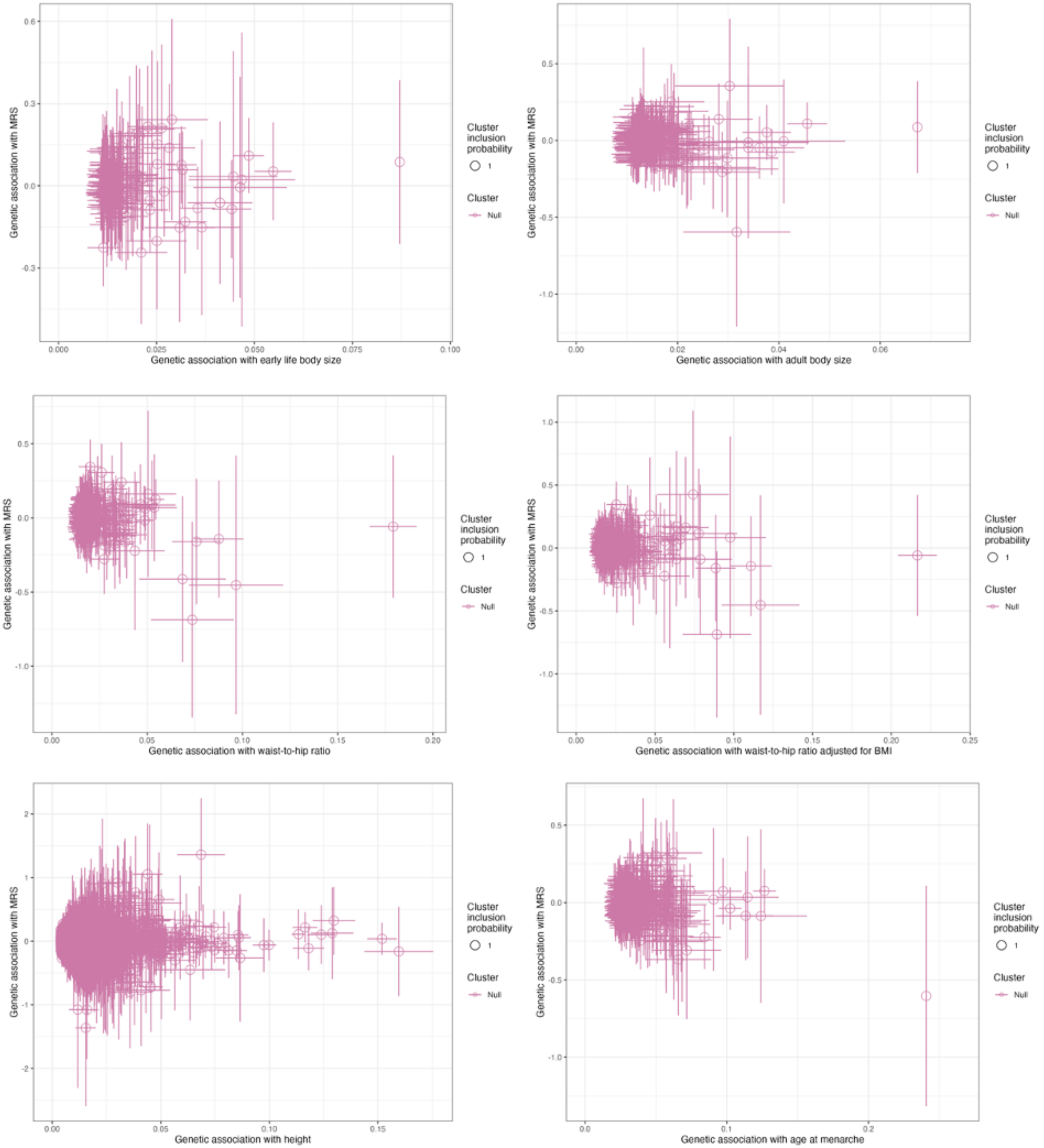


**Figure S5.** Genetic associations between each risk factor and mammogram risk score (MRS) using instrumental variable-MRS associations adjusted for predicted BI-RADS density. Scatter plots show results from MR-Clust analysis, with genetic association with MRS (y-axis) versus genetic association with each risk factor (x-axis). Each point represents a genetic variant, with lines indicating 95% confidence intervals.

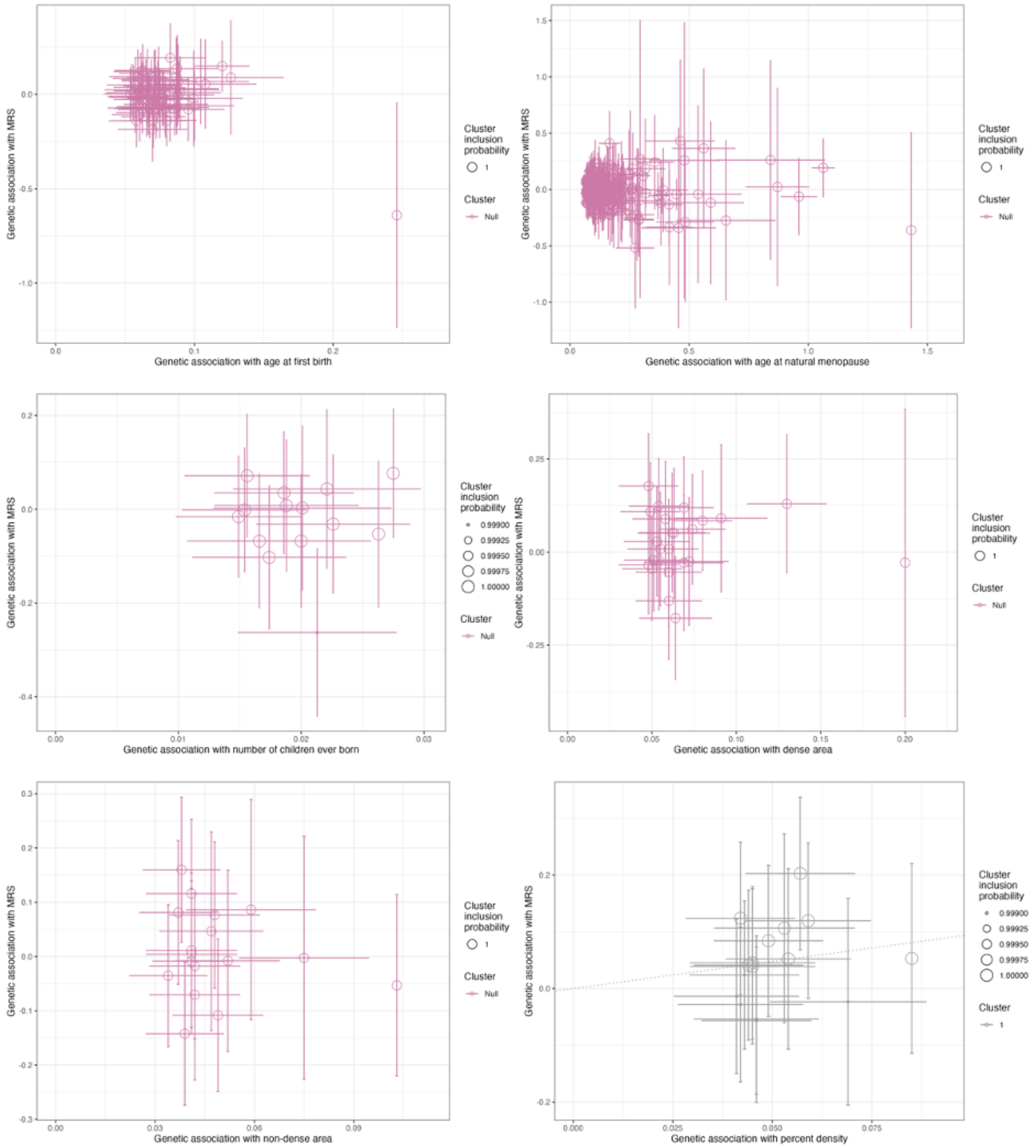


**Figure S5 (continued).** Genetic associations between each risk factor and mammogram risk score (MRS) using instrumental variable-MRS associations adjusted for predicted BI-RADS density. Scatter plots show results from MR-Clust analysis, with genetic association with MRS (y-axis) versus genetic association with each risk factor (x-axis). Each point represents a genetic variant, with lines indicating 95% confidence intervals.
